# Induction of Systemic Resistance against Aphids by Endophytic *Bacillus velezensis* YC7010 via Expressing *PHYTOALEXIN DEFICIENT4* in *Arabidopsis*

**DOI:** 10.3389/fpls.2017.00211

**Published:** 2017-02-15

**Authors:** Md. Harun-Or- Rashid, Ajmal Khan, Mohammad T. Hossain, Young R. Chung

**Affiliations:** Division of Applied Life Science (BK21 Plus), Plant Molecular Biology and Biotechnology Research Center, Gyeongsang National UniversityJinju, South Korea

**Keywords:** *Bacillus velezensis* YC7010, aphid, *Arabidopsis*, induced systemic resistance, *PAD4*

## Abstract

Aphids are the most destructive insect pests. They suck the sap and transmit plant viruses, causing widespread yield loss of many crops. A multifunctional endophytic bacterial strain *Bacillus velezensis* YC7010 has been found to induce systemic resistance against bacterial and fungal pathogens of rice. However, its activity against insects attack and underlying cellular and molecular defense mechanisms are not elucidated yet. Here, we show that root drenching of *Arabidopsis* seedlings with *B. velezensis* YC7010 can induce systemic resistance against green peach aphid (GPA), *Myzus persicae*. Treatment of bacterial suspension of *B. velezensis* YC7010 at 2 × 10^7^ CFU/ml to *Arabidopsis* rhizosphere induced higher accumulation of hydrogen peroxide, cell death, and callose deposition in leaves compared to untreated plants at 6 days after infestation of GPA. Salicylic acid, jasmonic acid, ethylene, and abscisic acid were not required to confer defense against GPA in *Arabidopsis* plants treated by *B. velezensis* YC7010. Bacterial treatment with *B. velezensis* YC7010 significantly reduced settling, feeding and reproduction of GPA on *Arabidopsis* leaves via strongly expressing senescence-promoting gene *PHYTOALEXIN DEFICIENT4* (*PAD4*) while suppressing *BOTRYTIS-INDUCED KINASE1* (*BIK1*). These results indicate that *B. velezensis* YC7010-induced systemic resistance to the GPA is a hypersensitive response mainly dependent on higher expression of *PAD4* with suppression of *BIK1*, resulting in more accumulation of hydrogen peroxide, cell death, and callose deposition in *Arabidopsis*.

## Introduction

Plants are usually challenged by various herbivorous insects in their natural environments. They have to develop diverse defense responses to protect themselves against attacks from different insects including aphids. Green peach aphid (GPA), *Myzus persicae*, a phloem sap feeding insect, has a wide range of hosts. It causes severe yield losses. It is also involved in the transmission of several plant viral diseases (Kennedy et al., 1962; Matthews, 1991; Blackman and Eastop, 2000). These aphids have highly modified stylets that enable them to enter sieve elements and secrete gelling and watery saliva to a certain extent during probing and feeding. Salivation of aphid plays a major role in successful colonization. It is also associated with its virulence (Tjallingii, 2006). During infestation of plants by aphids, the major defense related plant hormones are salicylic acid (SA), jasmonic acid (JA), ethylene (ET), and abscisic acid (ABA). They are reported to be involved in the induced systemic resistance (ISR) of many plants against aphids (Morkunas and Gabryś, 2011). The SA signaling pathway is activated to induce resistance in a number of plant species by aphid feeding (Moran et al., 2002; Zhu-Salzman et al., 2004; Coppola et al., 2013). On the contrary, Pegadaraju et al. (2005) have reported that SA signaling is not involved in the defense response of *Arabidopsis* against aphids. It has been reported that exogenous application of JA to a tomato plant can induce systemic defense against potato aphid (Cooper and Goggin, 2005). The population of GPA is increased in ET-insensitive *Arabidopsis* mutant *ein2*, indicating that ET can also confer resistance to aphid (Kettles et al., 2013). Resistance of *Arabidopsis* to aphid also depends on ABA biosynthesis and signaling (Kerchev et al., 2013).

Plant defense and cell death pathways induced by pathogens and insects are often regulated by certain plant hormones to elicit the accumulation of hydrogen peroxide (H_2_O_2_) and callose deposition (De Vos et al., 2005; Ahn et al., 2007; Zhou et al., 2009). In pathogen-infected plants, the production of reactive oxygen species (ROS, e.g., H_2_O_2_), and consequentially the onset of cell death referred to as ‘hypersensitive response’ (HR) can lead to systemic resistance (Durrant and Dong, 2004; Jones and Dangl, 2006; Singh et al., 2016). ROS and local cell death are also major defense mechanisms used by plants to protect themselves against phloem sap feeding GPA (Lei et al., 2014). *Arabidopsis PHYTOALEXIN DEFICIENT4* (*PAD4*) is especially crucial for its defense against aphids. Elevated expression of *PAD4* can deter GPA from settling on plants or feeding from the sieve elements (Louis et al., 2012; Lei et al., 2014). In addition, it has been reported that *PAD4* can stimulate premature leaf senescence, resulting in elevated expression of a subset of *SENESENCE ASSOCIATED GENES* (*SAG*) characterized by chlorophyll loss and cell death in GPA infested plants (Pegadaraju et al., 2005, 2007). The expression of *PAD4*-dependent constitutive expression of *SAG13* can confer hyper-resistance of *Arabidopsis* to GPA infestation (Louis et al., 2010). Recently, it has been reported that the molecular mechanism of *PAD4* resistance against aphid is reliant on interaction with *BOTRYTIS-INDUCED KINASE1* (*BIK1*). The mutant *bik1* can induce resistance to aphids through ROS production, cell death and leaf senescence. Such *bik1* induced resistance is dependent on the expression of *PAD4.* However, *BIK1* overexpression can make *Arabidopsis* plants more susceptible to aphid infestation (Lei et al., 2014). It has been shown that *BIK1* can control plant defense against aphids by negatively regulating *PAD4* expression (Louis and Shah, 2014).

*BOTRYTIS-INDUCED KINASE1*, a receptor-like cytoplasmic kinase (RLCK), is directly phosphorylated by BRASSINOSTEROID INSENSITIVE1-ASSOCIATED RECEPTOR KINASE (BAK1) and associated with FLAGELLIN-SENSITIVE2 (FLS2)/BAK1 complex in modulating pathogen-associated molecular patterns (PAMPs) mediated signaling (Lu et al., 2010; Zhang et al., 2010; Liu et al., 2013). Detection of PAMPs or microbe-associated molecular patterns (MAMPs) by particular transmembrane pattern recognition receptors is responsible for basal plant defense response collectively referred to as MAMP-triggered immunity (MTI) (Boller and Felix, 2009; Monaghan and Zipfel, 2012). The most characterized PAMP/MAMP receptors are receptor like kinases (RLKs), among which FLS2 and EF-TU RECEPTOR (EFR) can recognize bacterial elongation factor EF-Tu (Gómez-Gómez and Boller, 2000; Zipfel et al., 2006). Upon binding to their cognate MAMPs, FLS2, or EFR is associated with BAK1 (another RLK) during bacterial interaction with host (Chinchilla et al., 2007). For successful colonization of rhizobacteria and beneficial interaction with host plants, it is necessary to suppress MTI (Gutjahr and Paszkowski, 2009; Zamioudis and Pieterse, 2012). *Pseudomonas fluorescens* WCS417, one of plant growth promoting rhizobacteria (PGPR), has been shown to be able to suppress flagellin-triggered MTI responses and induce callose depositions during colonization in *Arabidopsis* (Millet et al., 2010). Callose deposition on sieve plates of rice plants can affect phloem transportation. It plays an important role in preventing brown planthopper (BPH) from ingesting the phloem sap (Hao et al., 2008). Several PGPR have been reported to use ISR to protect plant against pathogens. However, few studies have reported on ISR used by PGPR against insects (Zehnder et al., 1997; Lugtenberg and Kamilova, 2009; Pieterse et al., 2012). The main mechanisms of these bacteria involved in ISR upon pathogen infection or insect infestation include HR-type reactions, elevated cell wall or apoplastic peroxidase activity, callose deposition, and H_2_O_2_ accumulation (Conrath, 2006; Valenzuela-Soto et al., 2010; Niu et al., 2011; Rahman et al., 2015). Recently, these PGPR have been used for plant growth promotion, stress tolerance and biocontrol agents for insects and plant pathogens (Phi et al., 2010; Van de Mortel et al., 2012; Chung et al., 2015; Hossain et al., 2016; Zebelo et al., 2016). Some endophytic PGPR inhabiting the interior of host plants have shown ISR activity against insects (de Oliveira Araujo, 2015).

Recently, we have reported that novel endophytic strain of *B. oryzicola* YC7010 isolated from rice roots can inhibit the growth of important fungal and bacterial pathogens of rice such as *Fusarium fujikuroi* and *Burkholderia glumae* via antibiotic production and ISR (Chung et al., 2015; Hossain et al., 2016). The novelty of this species is now on debate and the name for this species was suggested to be changed as *B. velezensis* (Dunlap et al., 2016). The objective of this study was to determine whether *B. velezensis* YC7010 could induce systemic resistance against GPA in *Arabidopsis* and elucidate its underlying mechanism in terms of enhancing the expression of *PAD4* to activate cellular defense responses.

## Materials and Methods

### Plant Materials, Growth Conditions, and Aphid Rearing

Wild-type *Arabidopsis* ecotype Columbia-0 (Col-0), NahG and mutants *sid2, jar1, ein2-1, abi2-2, pad4, bik1*, and *bik1pad4* were used in this experiment. Seeds were sterilized with 70% (v/v) ethanol for 5 min followed by treatment with 1.2% (v/v) sodium hypochlorite (NaOCl) for 5 min. They were then washed with sterile distilled water. After sterilization, seeds were kept at 4°C for 48 h and grown on 0.5x Murashige and Skoog (MS) agar media supplemented with 1% (w/v) sucrose. Agar plates were then kept horizontally in a plant growth chamber for growing seedlings. Seedlings at 10 days old were transferred into pots containing 100 g soils autoclaved twice for 20 min at 120°C. Phloem sap-feeding GPA were cultured on cabbage (*Brassica oleracea*) and maintained in an environmental chamber with a long day photoperiod (16 h of light and 8 h of darkness) at 22°C with a light intensity of 100 μmol m^-2^ s^-1^. All experiments were performed in the growth chamber.

### Bioassay for Induced Systemic Resistance to Aphid by Bacteria

For bacterial inoculation of *Arabidopsis* plants, strain *B. velezensis* YC7010 (KACC18228, Jeonju, Korea) was cultivated in one-tenth strength tryptic soy broth (1/10 TSB, Bacto^TM^, Sparks, MD, USA) at 28°C for 48 h on a rotary shaker (160 rpm). Cells were then harvested by centrifugation at 6,000 × *g* for 15 min and suspended in a buffer solution (10 mM MgSO_4_) to adjust to 2 × 10^7^ colony forming unit (CFU) ml^-1^ for use. Seedlings of *Arabidopsis* at 4 weeks old were drenched with 10 ml bacterial suspension on the rhizosphere in each pot. Equal volume of 10 mM MgSO_4_ was drenched as control. No-choice and choice tests were performed to assess ISR to aphid after bacterial treatment. For the no-choice tests, five second-instar nymphs were placed at 5 days after bacterial inoculation. Seven days after infestation, total aphid population (adults and nymphs) on each plant was recorded. Each treatment had eight replicates. For the choice tests, 35 adult aphids were released at an equal distance between bacterial inoculated and uninoculated plants of different genotypes. At 24 h after releasing, the number of adult aphids settled on each plant was recorded. For each comparison, eight pairs of plants were used.

### Determination of Aphid Feeding Activity

Aphid feeding activity can be determined by measuring the amount of produced honeydew. To measure honeydew production, line split Whatman filter papers were placed under *Arabidopsis* plants of both treated and untreated plants infested by 30 adult aphids. To avoid absorbance of water from soil, a plastic membrane was placed under the filter paper. The filter papers used to collect honeydew at 3, 4, and 5 days after infestation of aphids were soaked in 0.1% (w/v) ninhydrin solution in acetone and dried in a 65°C oven for 30 min. Purple spots were shown when honeydew was stained by ninhydrin (Kim and Jander, 2007). To quantify honeydew stains, the stained filter papers were cut into pieces and extracted with 1 ml of 90% (v/v) methanol for 1 h at 4°C with continuous agitation. The absorbance of the supernatant was measured at 500 nm after centrifugation at 6,000 × *g* for 1 min. Methanol (90%) was used as a blank (Nisbet et al., 1994).

### Measurement of Hydrogen Peroxide Content

The concentrations of H_2_O_2_ in bacterial treated plants were measured at 0, 12, 24, 48, and 72 h after aphid infestation using a spectrophotometer. Treated leaf tissues (200 mg) were homogenized with 3 ml phosphate buffer (50 mM, pH 6.8) containing 1 mM hydroxylamine (catalase inhibitor). The mixture was centrifuged at 6,000 × *g* for 25 min and 3 ml of the supernatant was mixed with 1 ml 0.1% (v/v) titanium sulfate in 20% (v/v) H_2_SO_4_. The absorbance of the supernatant of the mixture was then determined at 410 nm after centrifugation at 6,000 × *g* for 15 min. Extinction co-efficient (0.28 μmol^-1^ cm^-1^) was used to calculate H_2_O_2_ content (Jana and Choudhuri, 1982).

### Histochemical Analyses of Cellular Defense Responses

For histochemical analyses of cellular defense responses, H_2_O_2_ accumulation, cell death, and callose deposition in *Arabidopsis* leaves were observed. Four weeks old *Arabidopsis* plants were treated with the same bacterial strain used for bioassay of ISR. A total of 40 aphids were placed on both treated plants and untreated control plants at 5 days after bacterial treatment. To visualize H_2_O_2_ accumulation, collected leaves at 6 days after infestation of aphids were vacuum infiltrated with 3,3’-diaminobenzidine (DAB) solution (1 mg ml^-1^ of DAB in pH 3.5 water). Under dark condition, leaves were immersed in the same solution overnight followed by destaining with 95% (v/v) ethanol until clear. They were preserved in 50% (v/v) ethanol. A dissecting microscope was used to capture the image of accumulated H_2_O_2_ in the leaves.

To visualize cell death of treated leaves, trypan blue staining was performed. Trypan blue was dissolved at a concentration of 0.125 mg ml^-1^ in lactophenol solution (phenol:lactic acid:glycerol:water [1:1:1:1]). Leaves were boiled in this staining solution for 1 min and destained in 95% (v/v) ethanol after cooling. Cell death images of leaves were captured with an Olympus Provis AX70 microscope at 10× magnifications.

To detect callose deposition, aniline blue staining was performed using published protocol (Clay et al., 2009). Buffer solution containing 10% (v/v) formaldehyde, 5% (v/v) acetic acid, and 50% (v/v) ethanol was used for fixation of *Arabidopsis* leaves at 37°C overnight. Fixed leaves were washed in 95% ethanol several times until clear, rinsed twice in water and then stained for 4 h or longer in the dark with 0.01% (w/v) aniline blue in 150 mM K_2_HPO_4_ (pH 9.5). Callose deposition was visualized with an Olympus Provis AX70 microscope at 10 × magnifications under UV illumination equipped with a broad band DAPI filter set. To quantify callose accumulation in the *Arabidopsis* leaves, images were subjected to intensity analysis using the image processing software IMAGEJ.

### Determination of Bacterial Population

In order to determine the bacterial population of *B. velezensis* YC7010 in *Arabidopsis* roots during plant growth, 10 ml of cell suspension (2.0 × 10^7^ CFU/ml) was drenched into *Arabidopsis* seedlings in a pot containing 100 g soils. Roots pieces (100 mg) were collected from treated seedlings at 0, 1, 2, 4, and 8 days after inoculation. Collected samples were surface-sterilized with 1.2% (v/v) NaOCl for 5 min followed by 70% ethanol treatment for 5 min. Finally, they were washed with sterile distilled water several times. The sterilized samples were homogenized in a buffer solution (10 mM MgSO_4_) using a sterile pestle and mortal. The aliquots were appropriately diluted before plating onto 1/10 TSB agar media supplemented with chloramphenicol (40 μgml^-1^) (Hossain et al., 2016). Plates were incubated at 28°C for 48 h. The CFU/g of fresh roots was counted.

### Measurement of Chlorophyll Content

To measure chlorophyll content, bacterial suspension of *B. velezensis* YC7010 and 40 aphids were used to treat 4 weeks old *Arabidopsis* plants using the protocols described earlier. Leaves were then collected at 6 days after aphid infestation of treated and untreated plants. Sample tissue (10 mg) was crushed with 1 ml of 80% (v/v) acetone in a glass grinder followed by centrifugation at 13,000 × *g* for 5 min. The absorbance of the supernatant was measured at wavelengths of 646.8 and 663.2 nm using a spectrophotometer. Total chlorophyll content was calculated using the following formula (Lichtenthaler, 1987): Total chlorophyll (mg/fresh weight or fw) = (7.15 ^∗^ A_663.2_ + 18.71 ^∗^ A_646.8_)/1000/fw of leaves.

### Quantitative RT-PCR

Bacterial suspension of *B. velezensis* YC7010 and 40 aphids were used to treat 4 weeks old *Arabidopsis* plants as described previously. Leaves were collected at 0, 12, 24, 48, and 72 h after aphid infestation of treated and untreated plants. Collected samples were frozen and ground in liquid nitrogen to a fine powder. Total RNA was extracted by using RNA extraction kit (Qiagen RNeasy Plant Mini Kit) and used to synthesis of complementary DNA using QuantiTect Reverse Transcription Kit according to the manufacturer’s instruction. Using SYBR Green Master Mix (Bio-Rad), quantitative reverse transcription RT-PCR reactions were performed according to the manufacturer’s protocol. Primers for candidate genes were designed using Primer3 software. Primer sequences are provided in Supplementary Table S1. To quantitatively determine the accumulation levels of genes, 2^-ΔΔCt^ method (Livak and Schmittgen, 2001) was used. Expression of genes was normalized against *Arabidopsis UBIQUITIN10* (AT4G05320) as an internal reference. All experiments were repeated three times and each real time PCR sample was run in triplicates.

### Statistical Analysis

All data were subjected to analysis of variance (ANOVA). Mean differences were estimated by Tukey’s honestly significant difference (HSD) using statistical software SPSS 17 (SPSS Inc., Chicago, IL, USA) and Sigma plot (version 12). Statistical significance was considered when *P* value was less than 0.05.

## Results

### *B. velezensis* YC7010 Induces Systemic Resistance to Aphids in *Arabidopsis*

**FIGURE 1:**
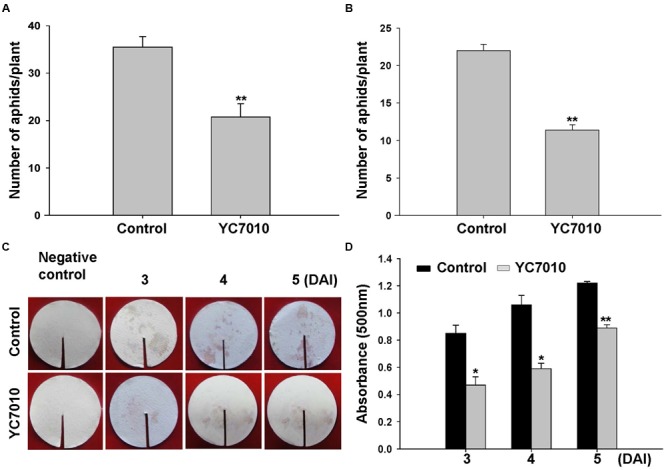
**Induction of systemic resistance to green peach aphids by treating *Arabidopsis* Col-0 plants with *B. velezensis* YC7010.** Bacterial suspension (2 × 10^7^ CFU/ml) was drenched to the rhizosphere of 4-weeks old *Arabidopsis* plants while 10 mM MgSO_4_ solution was used as negative control. **(A)** No-choice test. Five second-instar nymphs were placed at 5 days after bacterial inoculation and the total number of aphids on the leaves was counted at 7 days later after bacterial inoculation. **(B)** Choice test. The number of settled aphids was counted at 24 h after releasing 35 adults between two plants of the treated plants and control plants. **(C)** Effect of bacteria inoculation on aphid excretion of honeydew. The quantity of excreted honeydew was measured with intensity of ninhydrin stains. **(D)** Quantity of honeydew secretion was determined at optical density of 500 nm. DAI, days after infestation of aphids. Data were analyzed by independent Student’s *t*-tests. Bars represent mean values ± standard error (SE). Statistical significance for treatment effects is marked (^∗^*P* < 0.05; ^∗∗^*P* < 0.01). All experiments were conducted three times with similar results.

To determine whether *B. velezensis* YC7010 could induce resistance against aphids, both no-choice and choice tests were performed after drenching bacterial suspension to the rhizosphere of *Arabidopsis*. Such bacterial treatment significantly (*P* < 0.01) reduced the number of aphids than untreated control (**Figures 1A,B**). In the no-choice test, the number of aphids on bacterial treated plants was 20.75 per plant, which was significantly (*P* < 0.01) less than 35.50 on untreated plants. In the choice test, the number of aphids on bacterial treated plants was 11.38 per plant, which was also significantly (*P* < 0.01) lower than 22.00 on untreated plants. In the no-choice test and the choice test, the numbers of aphids on treated plants were reduced by 41.55 and 48.27%, respectively, by bacterial treatment when compared to those without bacterial treatment. In agreement with these results, aphids on bacterial treated plants showed significantly less excretion of honeydew, indicating less food intake at all time points at 3, 4 or 5 days after aphid infestation (**Figures 1C,D**).

### *B. velezensis* YC7010-Induced Systemic Resistance Is Dependent on Hydrogen Peroxide Production, Cell Death, and Callose Deposition in *Arabidopsis*

**FIGURE 2:**
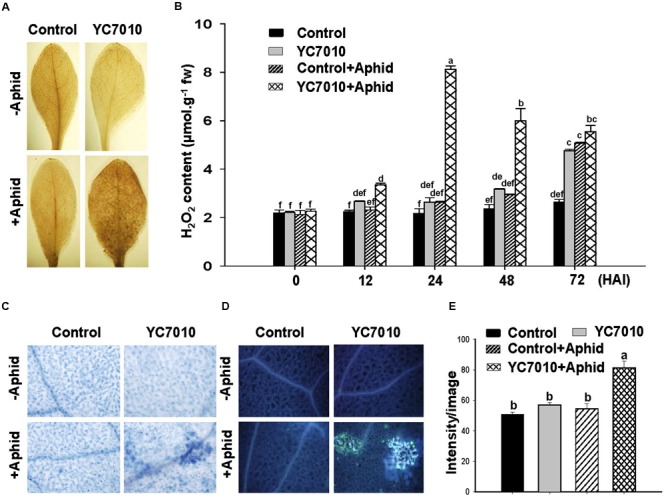
***B. velezensis* YC7010-mediated priming for activation of H_2_O_2_ accumulation, cell death, and callose deposition in the leaves of *Arabidopsis* Col-0 plants against aphid infestation.** Bacterial suspension (2 × 10^7^ CFU/ml) was used to treat 4-weeks old *Arabidopsis* plants and 40 aphids were placed at 5 days after bacterial treatment. **(A)** *Arabidopsis* leaves stained with 3,3’-diaminobenzidine, an indicator of H_2_O_2_, at 6 days after aphid infestation. **(B)** Content of H_2_O_2_ accumulated in the *Arabidopsis* leaves treated with bacteria at 0, 12, 24, 48, and 72 h after infestation (HAI) of aphids. **(C)** *Arabidopsis* leaves stained with trypan blue, a cell death indicator, at 6 days after aphid infestation. **(D)** Callose deposition in *Arabidopsis* leaves stained with aniline blue at 6 days after aphid infestation. **(E)** Quantification of the callose fluorescence on representative images using image intensity analysis with IMAGEJ software. Data were analyzed by one-way ANOVA. Means with different letters were significantly different (*P* < 0.05).

The effect of bacterial treatment on the accumulation of H_2_O_2_, cell death and callose deposition was observed at 6 days after aphid infestation. The accumulation of H_2_O_2_ and the level of H_2_O_2_ production at 12, 24, 48, and 72 h after infestation (HAI) of aphids in bacterial inoculated plants were significantly higher than those in untreated plants. The highest content of H_2_O_2_ was 8.12 μmol g^-1^ fw at 24 HAI in bacterial treated leaves. It was decreased to 6.00 μmol g^-1^fw at 48 HAI. However, this was still significantly higher than that in untreated plants after aphid infestation (**Figures 2A,B**). More cell death and callose deposition were detected in bacterial treated *Arabidopsis* plants comparing to those of untreated plants after aphid infestation (**Figures 2C–E**). A combination of bacterial treatment and aphid infestation led to significantly more H_2_O_2_ production, cell death and callose deposition, which might have contributed to effective defense response against aphids.

### *B. velezensis* YC7010-Induced Systemic Resistance Is Independent of SA, JA, ET, or ABA in *Arabidopsis*

**FIGURE 3:**
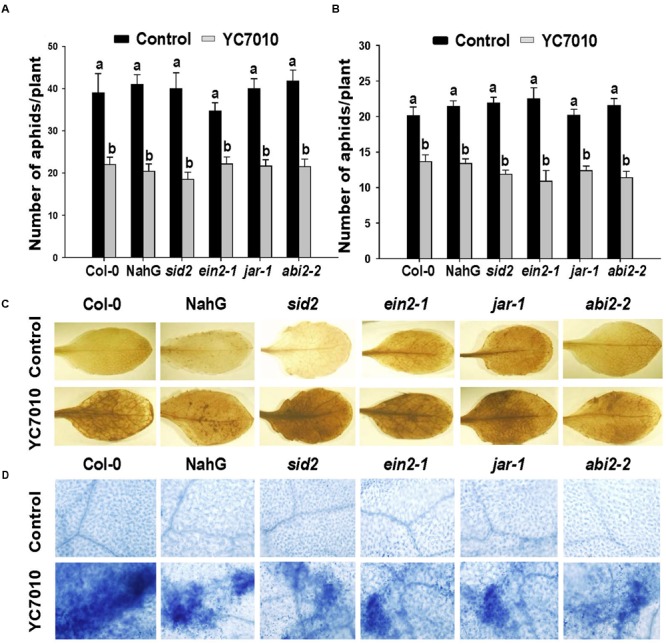
***B. velezensis* YC7010-induced systemic resistance to aphids, H_2_O_2_ accumulation and cell death in *Arabidopsis* plants were SA, JA, ET, and ABA independent. (A)** No-choice test on different genotypes. Five second-instar nymphs were placed at 5 days after bacterial inoculation and the total number of aphids on the leaves was counted at 7 days later after bacterial inoculation. **(B)** Choice test on different genotypes. The number of settled aphids was counted at 24 h after releasing 35 adults between two plants of the treated plants and control plants. **(C)** Representative leaf images of 3,3’-diaminobenzidine staining (H_2_O_2_ indicator). **(D)** Trypan blue staining (cell death indicator). Untreated control (top) or treated plants (bottom) at 6 days after aphids infestation. Data were analyzed by one-way ANOVA. Means with different letters were significantly different (*P* < 0.05).

To evaluate whether hormones such as SA, JA, ET, and ABA might play a role in *B. velezensis* YC7010 induced resistance to aphids, *Arabidopsis* plants such as wild ecotype Col-0, NahG, *sid2, jar1, ein2-1* and *abi2-2* were used in choice and no-choice tests. In both no-choice and choice tests, root drenching with bacterial suspension resulted in significant (*P* < 0.05) reduction in the number of aphids without significant difference among the Col-0, NahG and mutants (*sid2, jar1, ein2-1* or *abi2-2*) (**Figures 3A,B**). These results showed that *B. velezensis* YC7010 could confer resistance to aphids in all treated *Arabidopsis* plants regardless of mutation. Accumulation of H_2_O_2_ and cell death were also observed in all bacterial treated Col-0, NahG, and mutants. Both H_2_O_2_ accumulation and cell death were found in bacterial treated Col-0, NahG, *sid2, jar1, ein2-1*, or *abi2-2* plants. However, H_2_O_2_ accumulation and cell death were not detected in untreated control plants at 6 days after aphid infestation (**Figures 3C,D**). Collectively, these results indicate that the reduction in the number of aphids, H_2_O_2_ accumulation and cell death induced by *B. velezensis* YC7010 is not dependent on SA, JA, ET, or ABA in *Arabidopsis*.

### *B. velezensis* YC7010-Induced Aphid Resistance Is Dependent on Interactions among *PAD4, BIK1*, and *SAG13*

**FIGURE 4:**
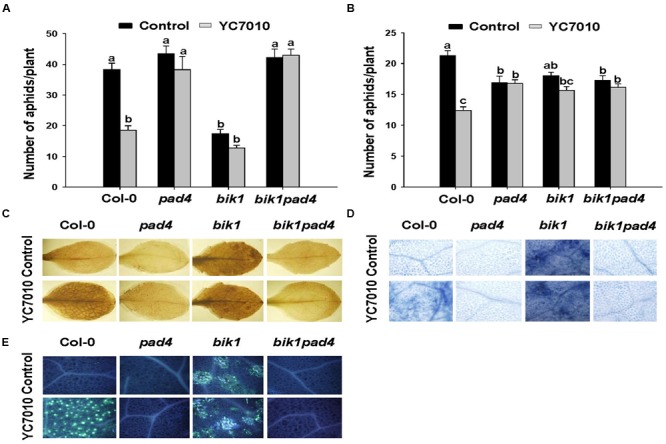
***B. velezensis* YC7010-induced systemic resistance to aphids, H_2_O_2_ accumulation, cell death, and callose deposition in *Arabidopsis* plants were *PAD4* dependent. (A)** No-choice test on different genotypes. Five second-instar nymphs were placed at 5 days after bacterial inoculation and the total number of aphids on the leaves was counted at 7 days later after bacterial inoculation. **(B)** Choice-test on different genotypes. The number of settled aphids was counted at 24 h after releasing 35 adults between two plants of the treated plants and control plants. **(C)** Representative leaf images of 3,3’-diaminobenzidine staining (H_2_O_2_ indicator). **(D)** Trypan blue staining (cell death indicator). **(E)** Callose deposition in *Arabidopsis* leaves stained with aniline blue. Untreated control (top) or treated plants (bottom) at 6 days after aphids infestation. Data were analyzed by one-way ANOVA. Means with different letters were significantly different (*P* < 0.05).

**FIGURE 5:**
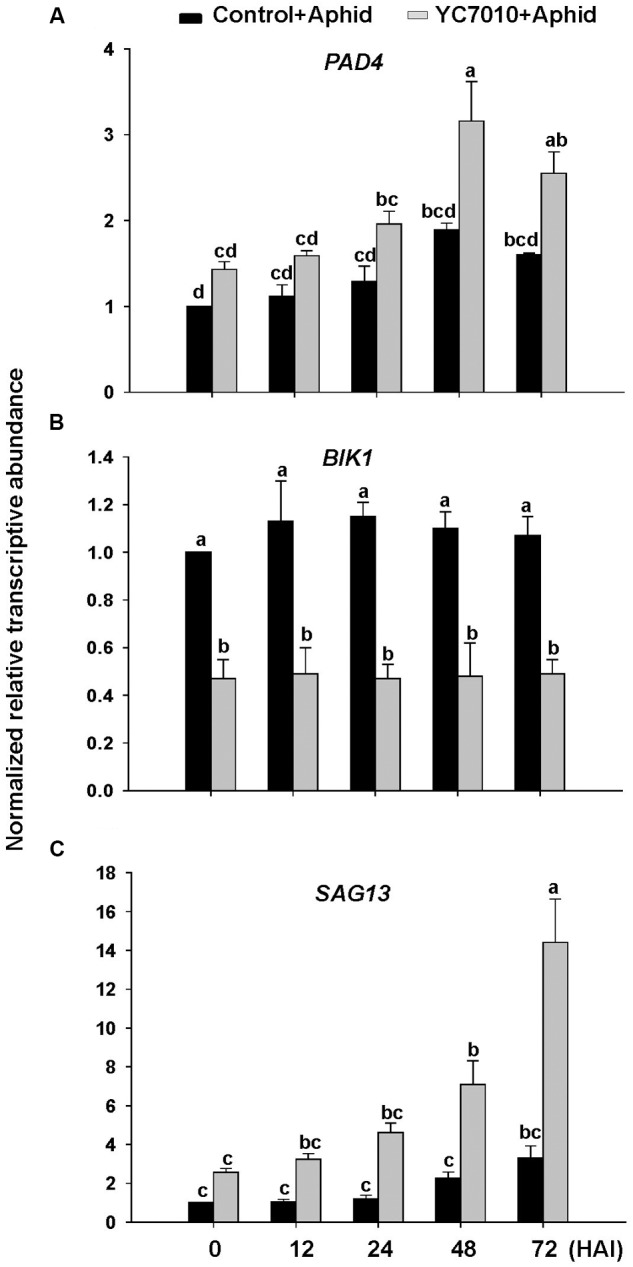
**Gene expression in leaves of *Arabidopsis* Col-0 plants after infestation with aphids.** Relative transcriptional levels of **(A)** *PAD4*, **(B)** *BIK1*, and **(C)** *SAG13*. *Arabidopsis* plants were treated with *B. velezensis* YC7010. At 5 days after treatment, 40 aphids were placed on both treated plants and control plants. Transcriptional levels were determined at 0, 12, 24, 48, and 72 h after infestation (HAI). Error bars represent standard error of mean. Each real time PCR sample was run in triplicates. Data were analyzed by one-way ANOVA. Means with different letters were significantly different (*P* < 0.05).

**FIGURE 6:**
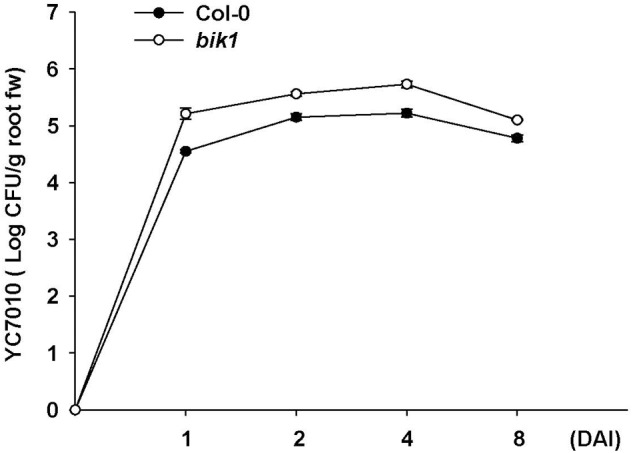
**Population of *B. velezensis* YC7010 colonized in the root of *Arabidopsis* Col-0 or *bik1* mutant plants.** The number of bacterial colonies grown on 1/10 TSB agar media supplemented with chloramphenicol (40 μg/ml) for 48 h at 28°C was counted using root pieces sampled at 0, 1, 2, 4, and 8 days after inoculation (DAI). Error bars represent standard error of mean.

To investigate whether *B. velezensis* YC7010-induced aphid resistance was dependent on *PAD4* associated with *BIK1*, we examined aphid performance on wild type Col-0 and mutants (*pad4, bik1*, and *bik1pad4*) of *Arabidopsis* treated with *B. velezensis* YC7010 (**Figure 4**). In both no-choice and choice tests, no significant difference in the number of aphids between bacterial treated and untreated all mutants was found. However, significantly less number of aphids was found on bacteria treated Col-0 *Arabidopsis* than that on untreated control *Arabidopsis* (**Figures 4A,B**). On the other hand, the number of aphids on bacterial treated or untreated *pad4* and *bik1pad4* mutants was higher than that on *bik1* (**Figure 4A**). Interestingly, in the no-choice test, aphid growth was suppressed in both treated and untreated *bik1* mutants. However, in the choice test, the number of aphids was not significantly different on these mutants regardless of bacterial treatment (**Figure 4B**). Accumulation of H_2_O_2_, cell death and callose were observed on bacterial inoculated Col-0, *bik1* and untreated *bik1* mutants after aphid infestation. However, none of these responses was detected on bacterial treated or untreated control *pad4* or *bik1pad4* mutants even with aphid infestation (**Figures 4C–E**). These results suggest that *PAD4* is required for H_2_O_2_ accumulation, cell death and callose deposition in *Arabidopsis*. More H_2_O_2_ accumulation, cell death and callose deposition were found on bacterial treated Col-0 and *bik1* plants treated with or without *B. velezensis* YC7010. Therefore, we investigated the expression patterns of *PAD4* and *BIK1* genes in Col-0 plants. At every time point, the expression level of *PAD4* was higher in *B. velezensis* YC7010 treated plants than that in untreated plants (**Figure 5A**). The highest expression level of *PAD4* was found in bacterial treated plants at 48 HAI of aphids. On the contrary, the expression level of *BIK1* was higher in untreated plant compared to that in bacterial treated plants with aphid infestation (**Figure 5B**). As *PAD4* can stimulate premature leaf senescence in aphids-infested *Arabidopsis* plants, we investigated whether the expression level of senescence associated *SAG13* gene was affected by *B. velezensis* YC7010 in Col-0 plants. The expression level of *SAG13* was higher in bacterial treated plants than in untreated plants at all time points after aphid infestation (**Figure 5C**). The highest expression was found in bacterial treated plants at 72 HAI of aphids. Lower chlorophyll content was found in most bacterial treated plants (except *pad4, bik1*, and *bik1pad4*) compared to that in untreated control Col-0 plants (Supplementary Figure S1). The contents of chlorophyll in the leaves of *pad4, bik1*, and *bik1pad4* mutants in treated and untreated plants were similar to each other. The chlorophyll content in *bik1* mutant was lower than that in *pad4* or *bik1pad4* mutant (treated or untreated), although the difference was not statistically significant. Bacterial count in the roots of *bik1* mutant was higher than that of Col-0 (**Figure 6**), indicating that root colonization of *B. velezensis* YC7010 was suppressed by *BIK1* in *Arabidopsis* plants.

## Discussion

*Arabidopsis* roots colonized by certain beneficial PGPR can activate ISR response that is effective against a broad range of plant pathogens (Van Oosten et al., 2008; Van Wees et al., 2008; Van der Ent et al., 2009). Likewise, some non-pathogenic rhizobacteria or endophytic bacteria commonly found inside roots also can enhance plant resistance against insect pests (Pineda et al., 2010; de Oliveira Araujo, 2015). These bacteria with ability to enhance plant growth and development have the potential to be utilized for biological control of numerous insect pests. We have previously identified an endophytic *B. velezensis* YC7010 with anti-microbial, plant growth-promoting and systemic resistance-inducing activities (Chung et al., 2015). In this study, our results demonstrated that root drenching of *Arabidopsis* with *B. velezensis* YC7010 suspension resulted in the establishment of an ISR against GPA, regardless of test methods (choice or no-choice tests, **Figure 1**). Up to date, few reports have been published on tritrophic interactions among bacteria, insects and host plants. Reports on ISR by endophytic bacteria is especially limited. Van de Mortel et al. (2012) have reported that colonization of *Arabidopsis* roots by non-pathogenic rhizobacteria can induce resistance against lepidopteran insect herbivore *Spodoptera exigua*, in agreement with our results. Less honeydew excretion by GPA also indicates less food intake from bacterial treated plants.

Inoculation of *B. velezensis* YC7010 enhanced H_2_O_2_ production, cell death, and callose deposition with aphid infestation in *Arabidopsis* (**Figure 2**). This indicates that ISR by this bacterial strain might be due to cellular responses, resulting in early cell death and callose deposition as HR. Insect feeding induced oxidative stress is an important component of plant defense to attacking insects. ROS detoxification may decrease antioxidant levels but increase toxic oxidation products in corn earworm infested soybean plants (Bi and Felton, 1995). Increased level of H_2_O_2_ and other oxidative products of ROS in plants can directly damage the midgut of insects and inhibit their growth. High mortality of insects by consumption of artificial diets containing H_2_O_2_ also supports the effect of ROS on the suppression of GPA (Liu et al., 2010). Another study has shown that higher level of H_2_O_2_ accumulation in rice (*Oryza sativa*) can enhance the resistance against phloem sap sucking BPH (*Nilaparvata lugens*) (Zhou et al., 2009). In addition, cellular accumulation of H_2_O_2_ can lead to plant cell death which may act as HR to GPA (Hoeberichts and Woltering, 2003). Cell death is considered as a plant defense factor against aphid by manipulating host nutritional quality in plant–microbe interactions (Goggin, 2007). Callose deposition is also an important defense mechanism that prevents insects from ingesting phloem sap (Hao et al., 2008). Microbes-mediated ISR is often associated with accumulation of H_2_O_2_, cell death, and deposition of callose in plant–pathogen interactions (Conrath et al., 2002; Jacobs et al., 2011). However, no such report has been published on three-way interaction of rhizobacteria (*B. velezensis* YC7010), host plants and aphids through ISR associated with these defense responses. It is possible that H_2_O_2_-induced cell death in infested and adjacent cells could limit photoassimilates flow to the feeding sites, which can move aphids away from their feeding sites. No discrete accumulation of H_2_O_2_ and callose deposition was detected in bacterial treated or untreated leaves before aphid infestation. However, accumulation of H_2_O_2_ and callose deposition were observed in leaves infested with GPA 6 days later. On the other hand, more H_2_O_2_ accumulation was found only in bacterial treated plants with GPA infestation at different time points (**Figure 2**). Similarly, accumulation of H_2_O_2_ and callose deposition was observed in *Arabidopsis* treated with rhizobacteria or inoculated by pathogens (Ahn et al., 2007). Primed plants show faster and/or stronger activation of cellular defenses when subsequently challenged by pathogen or insect attack, resulting in enhanced level of resistance (Conrath, 2011). These results suggest that *B. velezensis* YC7010 can induce priming responses against GPA in *Arabidopsis*, which is partially similar to results of previous studies, supporting the perception that ISR by beneficial microbes is commonly based on defense priming (Pieterse et al., 2014).

Our results showed that *B. velezensis* YC7010-mediated ISR to aphids were not dependent on SA, JA, ET, or ABA that were not associated with H_2_O_2_ accumulation or cell death in bacterial treated *Arabidopsis* plants (**Figure 3**). In other previous studies on aphid defense mechanisms, SA is not essential, while JA, ET, and ABA are not mainly involved in defense of *Arabidopsis* plants against aphid either (Pegadaraju et al., 2005; Lei et al., 2014). On the contrary, *PAD4* was found to be required for induction of *B. velezensis* YC7010-mediated resistance to aphid in this study. In addition, root drenching with *B. velezensis* YC7010 enhanced the expression of *PAD4* in *Arabidopsis* plants after aphid infestation (**Figures 4** and **5A**). Aphid feeding is well known to induce the expression of *PAD4* which is required for cell death-mediated resistance. It has been reported that transgenic plants with overexpression of *PAD4* can enhance their resistance against GPA more than wild type *Arabidopsis* Col-0 (Pegadaraju et al., 2005, 2007). In this study, the loss of *BIK1* function promoted the induced resistance against aphid in the no-choice test without bacterial treatment. HRs such as H_2_O_2_ accumulation and cell death were observed in both bacterial treated and untreated plants. However, they were absent in *bik1pad4* mutant, suggesting that *BIK1* might not directly repress, but indirectly modulate cell death pathway through *PAD4*. Root drenching with *B. velezensis* YC7010 suppressed the expression of MTI related gene *BIK1* (**Figure 5B**). However, the number of *B. velezensis* YC7010 in the root was higher in *bik1* mutant than that in wild type Col-0, indicating that suppression of *BIK1* might contribute to stable colonization of *B. velezensis* YC7010 (**Figure 6**). Similar to our results, it has been reported that root inoculation with *Bacillus cereus* AR156 can actively block immune responses in *Arabidopsis* roots in order to establish a compatible interaction with the host which is important for root colonization by bacteria (Niu et al., 2011). Lei et al. (2014) have also shown that *PAD4* expression is much higher in *bik1* mutant to suppress aphids. These results collectively demonstrate that root drenching of *B. velezensis* YC7010 can effectively suppress the growth of aphids in leaves via expression of *PAD4* which depends on the suppression of *BIK1* in *Arabidopsis*.

The colonization of *Arabidopsis* roots by *B. velezensis* YC7010 resulted in enhanced expression level of *SAG13* as well as chlorophyll loss in leaves upon aphid infestation (**Figure 5C**; Supplementary Figure S1). Senescence-associated processes have negative effect on aphid growth. For example, a gall aphid induced premature senescence in *Pistacia palaestina* trees has been shown to be correlated with reduced performance of another aphid feeding on the same leaflet (Inbar et al., 1995). However, *PAD4* stimulates the premature senescence of leaves which can confer resistance to aphids (Pegadaraju et al., 2005). These results suggested that root drenching of *B. velezensis* YC7010 can elevate the expression of *PAD4* and activate premature leaf senescence which is involved in resistance to aphid.

In summary, *B. velezensis* YC7010 root treatment could induce primed systemic resistance against GPA in *Arabidopsis*. Root colonization of *Arabidopsis* by *B. velezensis* YC7010 suppressed the expression level of *BIK1*, resulting in higher expression level of *PAD4* and *SAG13* in bacterial treated plants. Enhanced expression of *PAD4* triggered more rapid H_2_O_2_ accumulation, cell death, and callose deposition in bacterial treated *Arabidopsis* than in untreated plants after aphid infestation. To the best of our knowledge, this is the first report on the mechanism of ISR against aphid by an endophytic PGPR. In this aspect, the results of this study will be helpful for developing environmental friendly management strategies for insect pests using beneficial endophytic bacteria.

## Author Contributions

This study was designed by YC, MR, AK, and MTH. All experiments in this study were performed by MR. Data was analyzed by MR, AK, and MTH. The manuscript was written by YR and MR.

## Conflict of Interest Statement

The authors declare that the research was conducted in the absence of any commercial or financial relationships that could be construed as a potential conflict of interest.
