# Supplementary figures and images for: Induction of Systemic Resistance against Aphids by Endophytic Bacillus velezensis YC7010 via Expressing PHYTOALEXIN DEFICIENT4 in Arabidopsis
Source: Front Plant Sci. 2017 Feb 15;8:211. doi: 10.3389/fpls.2017.00211 (PMC5309228; doi:10.3389/fpls.2017.00211)

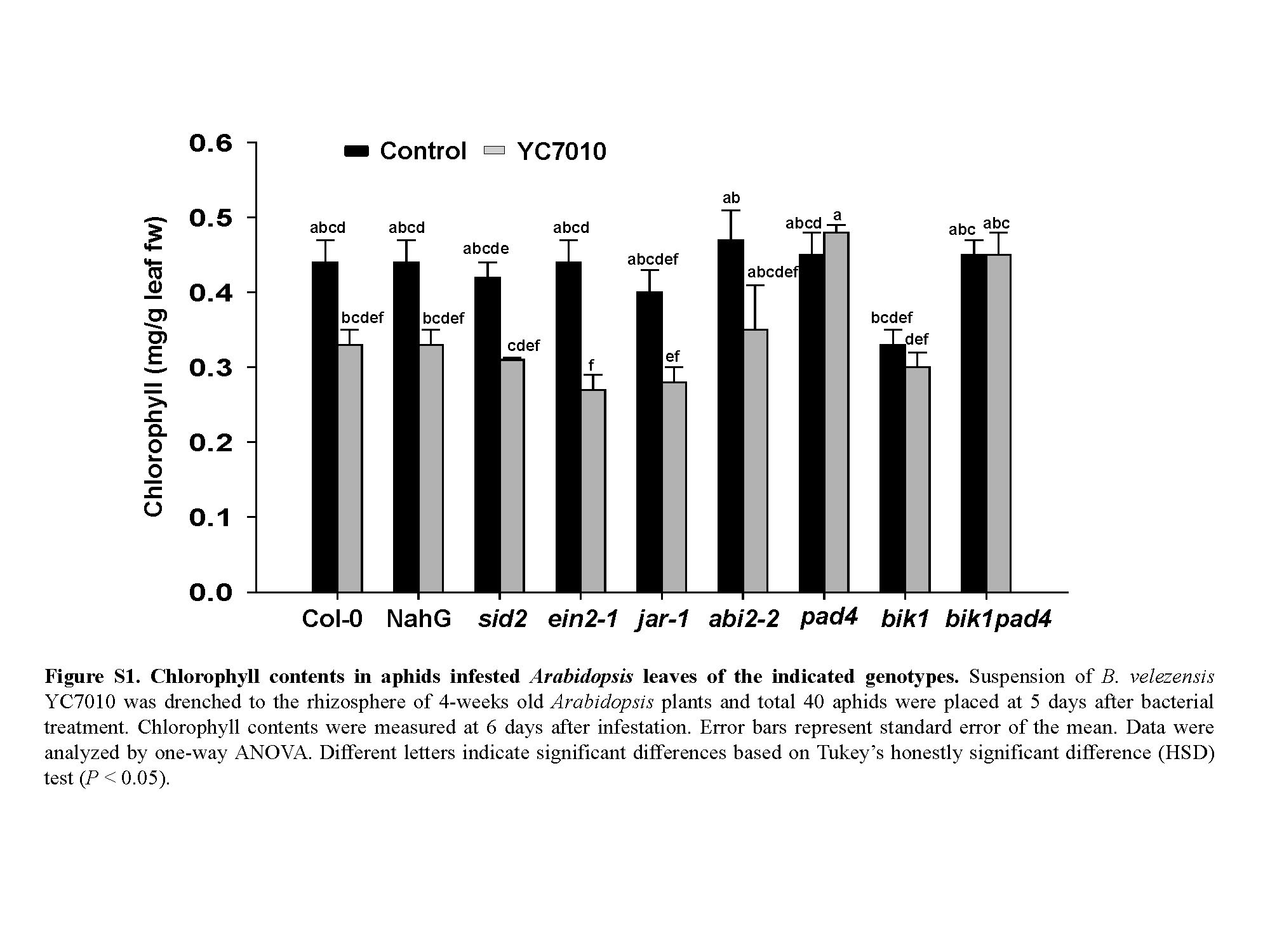

Supplement: Supplementary file 1 [file Image_1.JPEG]
