# Supplementary material for: Induction of Systemic Resistance against Aphids by Endophytic Bacillus velezensis YC7010 via Expressing PHYTOALEXIN DEFICIENT4 in Arabidopsis
Source: Front Plant Sci. 2017 Feb 15;8:211. doi: 10.3389/fpls.2017.00211 (PMC5309228; doi:10.3389/fpls.2017.00211)
Supplement: Supplementary file 2 [file Table_1.DOCX]

**Table S1. Primers used in this study for quantitative RT-PCR**

| Gene name | Accession  number | Forward primer (5' - 3') | Reverse primer (5' - 3') |
| --- | --- | --- | --- |
| *PAD4* | AT3G52430 | TCTTCAGTTAAAGATCAAGGAAGG | GGTTGAATGGCCGGTTATC |
| *SAG13* | AT2G29350 | GCCCACCCATTGTTAAAAGC | ACGACTCCAGCAGCAGAGGAT |
| *BIK1* | AT2G39660 | ACCGTCTTCTAGTCTACGAG | ATTGGACCGTCTCTAGCTAG |
| *UBQ10* | AT4G05320 | AGATCCAGGACAAGGAAGGTATTC | CGCAGGACCAAGTGAAGAGTAG |
